# Epidemiology of infectious diarrhoea and the relationship with etiological and meteorological factors in Jiangsu Province, China

**DOI:** 10.1038/s41598-019-56207-2

**Published:** 2019-12-20

**Authors:** Xinyu Fang, Jing Ai, Wendong Liu, Hong Ji, Xuefeng Zhang, Zhihang Peng, Ying Wu, Yingying Shi, Wenqi Shen, Changjun Bao

**Affiliations:** 1 School of Public Health, Nanjing Medical University, Nanjing, 211166 China; 2 Jiangsu Provincial Center for Disease Control and Prevention, Nanjing, 210009 China; 3 NHC Key laboratory of Enteric Pathogenic Microbiology, Nanjing, 210009 China

**Keywords:** Climate change, Ecological epidemiology, Bacterial infection, Viral infection, Risk factors

## Abstract

We depicted the epidemiological characteristics of infectious diarrhoea in Jiangsu Province, China. Generalized additive models were employed to evaluate the age-specific effects of etiological and meteorological factors on prevalence. A long-term increasing prevalence with strong seasonality was observed. In those aged 0–5 years, disease risk increased rapidly with the positive rate of virus (rotavirus, norovirus, sapovirus, astrovirus) in the 20–50% range. In those aged > 20 years, disease risk increased with the positive rate of adenovirus and bacteria (*Vibrio parahaemolyticus*, *Salmonella*, *Escherichia coli*, *Campylobacter jejuni*) until reaching 5%, and thereafter stayed stable. The mean temperature, relative humidity, temperature range, and rainfall were all related to two-month lag morbidity in the group aged 0–5 years. Disease risk increased with relative humidity between 67–78%. Synchronous climate affected the incidence in those aged >20 years. Mean temperature and rainfall showed U-shape associations with disease risk (with threshold 15 °C and 100 mm per month, respectively). Meanwhile, disease risk increased gradually with sunshine duration over 150 hours per month. However, no associations were found in the group aged 6–19 years. In brief, etiological and meteorological factors had age-specific effects on the prevalence of infectious diarrhoea in Jiangsu. Surveillance efforts are needed to prevent its spread.

## Introduction

Diarrhoea kills about 525,000 children under 5 years each year worldwide^1^. Infectious diarrhoea remains a substantial public health problem, particularly in developing countries. In China, it has been listed as a legal Class C infectious disease and also the second leading notifiable disease with an incidence of 93.10 per 100,000^2^. This disease occurs in all age groups, especially in children under 5 years.

Infectious diarrhoea morbidity varies temporally and spatially. For example, it peaks in December-January in Guangdong province^3^, August in Zhejiang province^4^, and July-August and November-December in Shanxi province^5^. The annual incidence in each region also differs. However, previous studies did not analyse the factors influencing infectious diarrhoea in their respective areas. Jiangsu Province, which is located in the eastern coastal areas of China, has shown an increasing incidence of infectious diarrhoea^6^. It is therefore necessary to explore the epidemiological characteristics and the factors influencing this trend to inform targeted prevention and control.

Age-specific morbidity of infectious diarrhoea could be related to pathogen type and climate. In particular, infectious diarrhoea is mainly caused by bacteria and viruses, which predominate differently in diverse populations and the prevalence of pathogens may influence age-specific morbidity differently^7,8^. Studies have shown that meteorological factors exert different lag effects on diarrhoea in different regions^9–11^. Climate may also influence human behaviour and epidemics of pathogens^12,13^, which may further influence the prevalence of diarrhoea. Wei *et al*. found that climate was differentially associated with diarrhoea morbidity in various age groups in Taiwan^9^. Nonetheless, to date, no study has investigated the lagged relationship between climate and age-specific morbidity nor quantified the age-specific effect of etiological and meteorological factors on infectious diarrhoea.

In this study, we first illustrated the epidemiological characteristics of infectious diarrhoea in Jiangsu Province from 2013 to 2017 and further explored the potential lagged relationship between the prevalence of pathogens, climate, and infectious diarrhoea in various populations. Finally, we applied generalized additive models (GAM) to quantify the age-specific effect of etiological and meteorological factors that are strongly related to infectious diarrhoea.

## Results

### Epidemiology of infectious diarrhoea

A total of 86,885 cases of infectious diarrhoea (representing a morbidity of 21.83/100,000 per year) were reported during 2013–2017 in Jiangsu. Cases aged 0–2 years accounted for 54.93% (47725/86885) of all cases. The morbidity in males (25.07/100,000) was higher than that in females (18.55/100,000) (χ^2^ = 1939.869, *P* < 0.01), with a ratio of 1.35:1.

**Figure 1:**
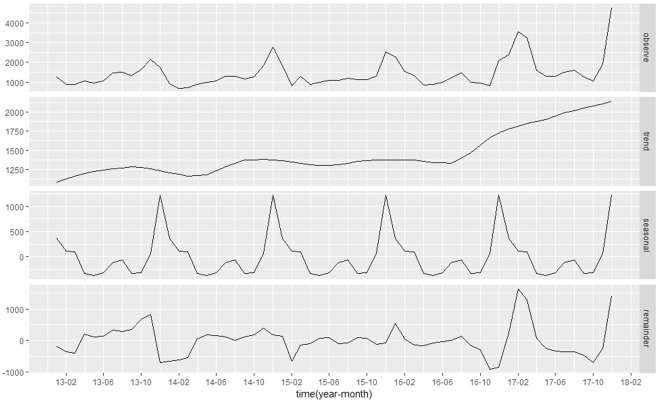
Monthly observed cases of infectious diarrhoea in Jiangsu Province, 2013–2017. Note: From top to bottom, the lines represent actually observed cases, trend components, seasonal components, and random components, respectively.

**Figure 2:**
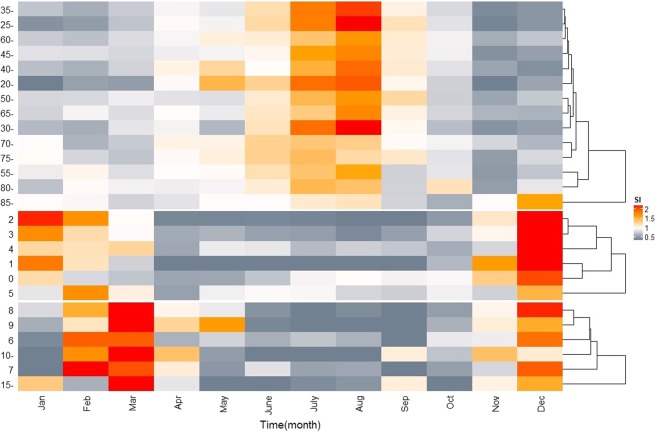
Seasonal index of age-specific infectious diarrhoea incidence in Jiangsu Province, 2013–2017.

The morbidity showed a long-term increasing trend. Two peaks were observed annually; a higher peak in November to January, and a relatively lower peak from July to September (Fig. 1). The population was clustered into three age groups: 0–5 years age group, 6–19 years age group, and >20 years age group. The incidence of infectious diarrhoea in the groups aged 0–5 years and 6–19 years increased mainly in the winter and spring and peaked in December in the group aged 0–5 years and December and March in the group aged 6–19 years. In comparison, morbidity peaked in July-August in those aged over 20 years (Fig. 2).

**Figure 3:**
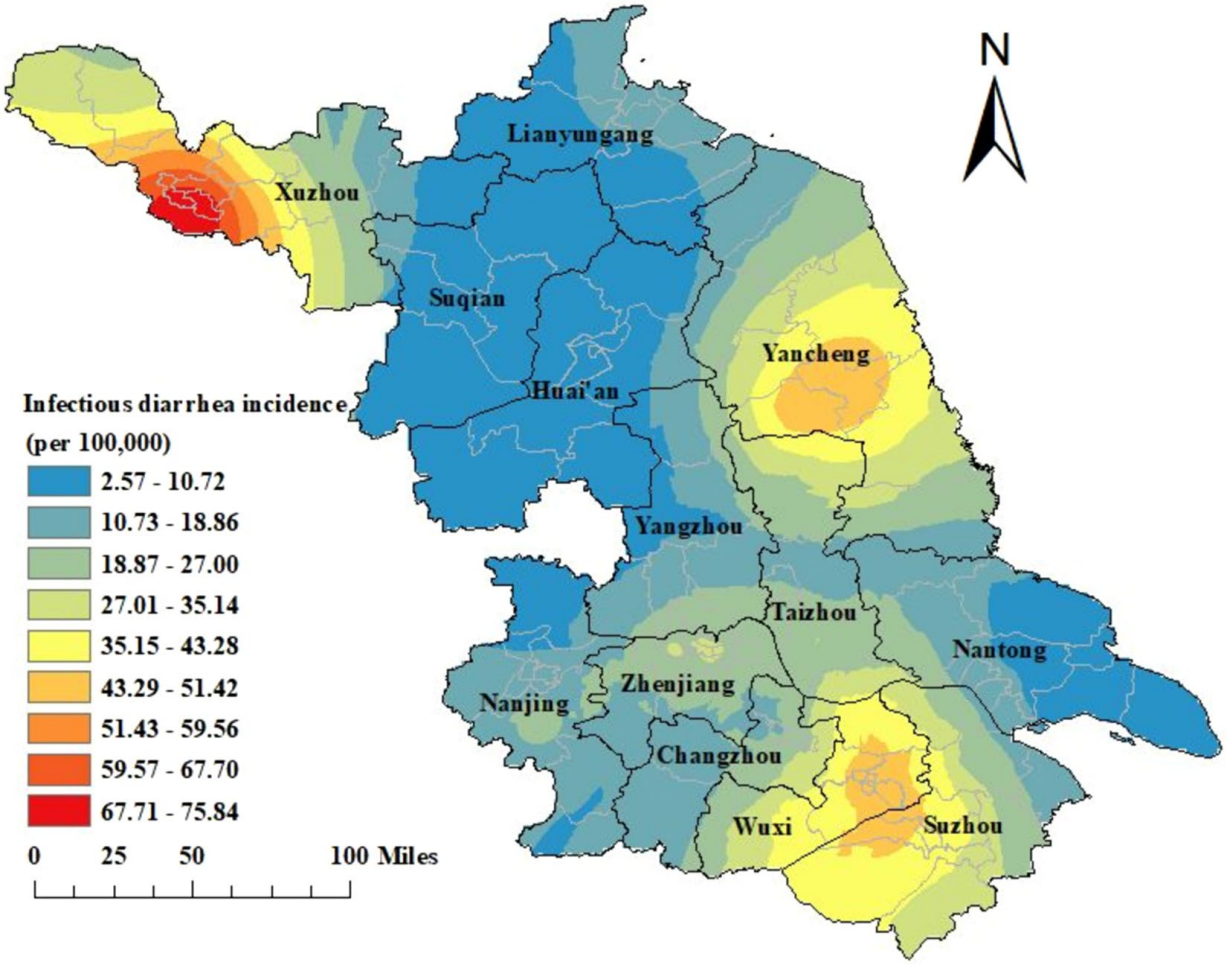
Smoothed map of infectious diarrhoea incidence in Jiangsu Province, 2013–2017.

Three high endemic regions were observed during 2013–2017. These were Xuzhou, followed by Yancheng and the border area between Wuxi and Suzhou. The incidence ranged from 0.92 to 155.39 per 100,000 at the county level. Yunlong district in Xuzhou had the highest incidence (155.39 per 100,000) (Fig. 3).

### Aetiology and climate characteristics

**Figure 4:**
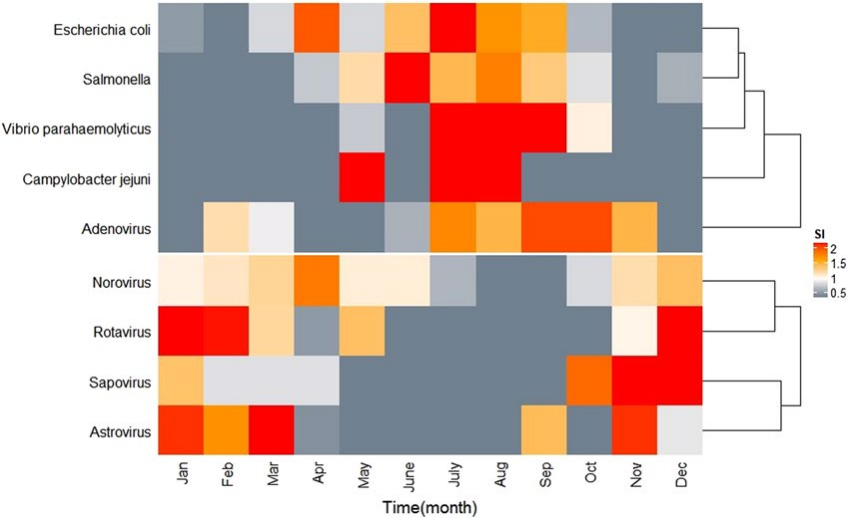
Seasonal index of the pathogens of infectious disease in Jiangsu Province.

A total of 1193 positive specimens were detected out of 6640 specimens resulting in a positive rate of 17.97% (1193/6640). The virus positive rate was 14.43% (958/6640) and was mainly from norovirus (7.52%) and rotavirus (5.36%). The bacterium positive rate was 3.95% (262/6640) and was mainly from *Salmonella* (1.82%) and *Escherichia coli*, (1.46%). The pathogens were clustered into two classes by their seasonality: Class 1 (adenovirus, *Vibrio parahaemolyticus*, *Salmonella*, *Escherichia coli*, *Campylobacter jejuni*) and Class 2 (rotavirus, norovirus, sapovirus, astrovirus). Class 1 pathogens were mainly prevalent from June to September and Class 2 pathogens from November to March (Fig. 4).

Basic information on meteorological factors are shown in the supplementary file. Latent multicollinearity was detected between mean temperature and rainfall (r_s_ = 0.80, *P* < 0.01), relative humidity and rainfall (r_s_ = 0.76, *P* < 0.01) and relative humidity and temperature range (r_s_ = −0.86, *P* < 0.01) (Table 1).

**Table 1:** Spearman correlation coefficients between meteorological factors.

Variables	Mean temperature	Relative humidity	Temperature range	Sunshine duration	Rainfall
Mean temperature	1				
Relative humidity	0.51*	1			
Temperature range	−0.33	−0.86*	1		
Sunshine duration	0.41	-0.31	0.40	1	
Rainfall	0.80*	0.76*	−0.61*	−0.01	1

Note: *Statistically significant at the 0.05 level (*P* < 0.05).

### Cross-correlation analyses

The prevalence of Class 2 pathogens positively correlated with disease morbidity in the group aged 0–5 years (r_s_ = 0.47, *P* = 0.01), and the prevalence of Class 1 pathogens positively correlated with disease morbidity in the group aged over 20 years (r_s_ = 0.43, *P* < 0.01). Nonetheless, no correlation was observed in the group aged 6–19 years.

The mean temperature, relative humidity, and rainfall were all positively related to the morbidity by two-month lag in the group aged 0–5 years, while there was a negative correlation with temperature range. Rainfall negatively correlated with the incidence in the 0–5 age group, but positively correlated with two-month lag incidence. Notably, disease morbidity in those aged over 20 years demonstrated strong positive correlation with mean temperature, sunshine duration, and rainfall as well as two-month-lag negative correlation with relative humidity. There was no correlation between meteorological factors and disease morbidity in the group aged 6–19 years (Table 2).

**Table 2:** Spearman correlation coefficients between meteorological factors and infectious diarrhoea morbidity in different age groups.

Variables	Mean temperature	Relative humidity	Temperature range	Sunshine duration	Rainfall
**0–5 years age group incidence**					
Lag0	−0.22	−0.20	0.04	−0.06	−0.38*
Lag1	0.07	0.15	−0.14	−0.02	−0.06
Lag2	0.35*	0.46*	−0.26*	0.03	0.32*
**6–19 years age group incidence**					
Lag0	−0.03	−0.17	0.14	0.15	−0.15
Lag1	0.01	0.06	−0.01	−0.09	0.06
Lag2	0.08	0.19	−0.13	−0.08	0.04
**Over 20 years age group incidence**					
Lag0	0.72*	0.25	−0.21	0.46*	0.51*
Lag1	0.54*	−0.05	0.04	0.43*	0.36*
Lag2	0.19	−0.28*	0.20	0.25	0.16

Note: Lag 0/1/2 represented the lag effects of zero to two months. *Statistically significant at the 0.05 level (*P* < 0.05).

### GAM results

**Figure 5:**
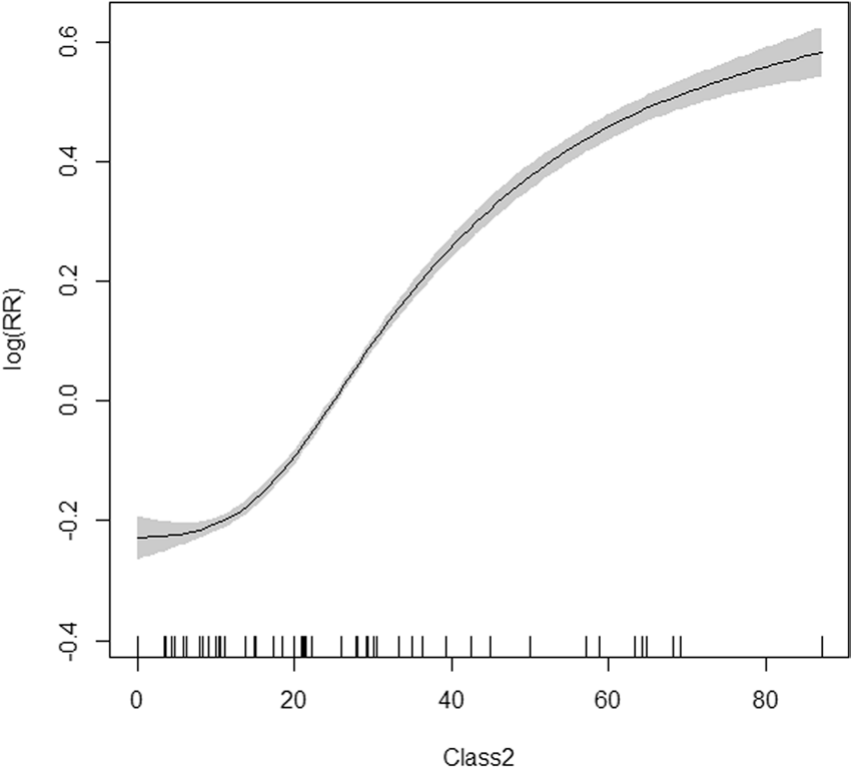
GAM model plot for the prevalence of Class 2 pathogens with infectious diarrhoea in the group aged 0–5 years.

**Figure 6:**
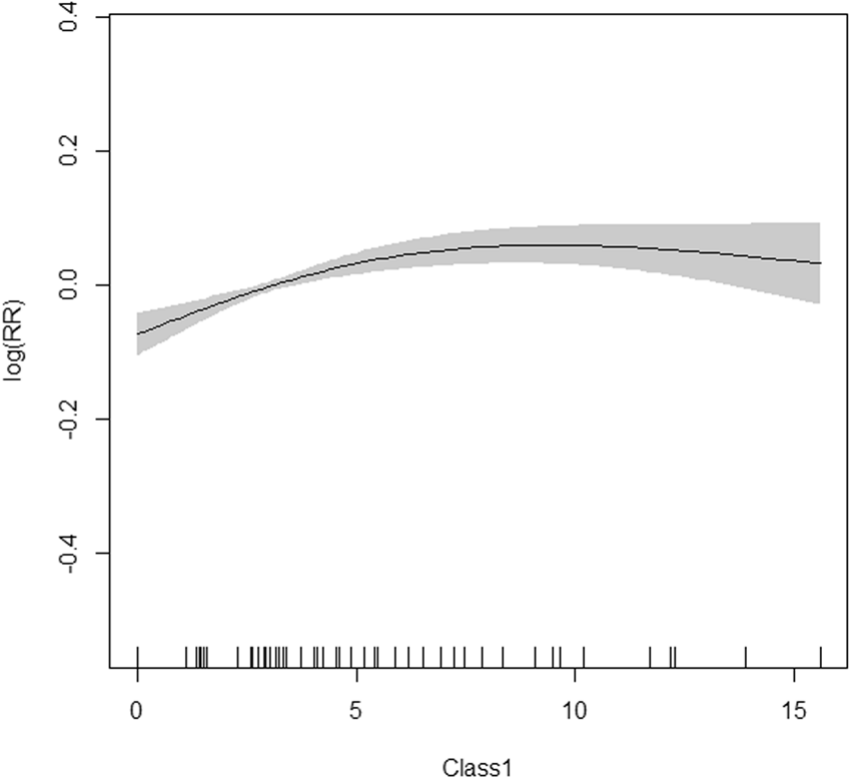
GAM model plot for the prevalence of Class 1 pathogens with infectious diarrhoea in the group aged over 20 years.

Etiological factors (the prevalence of Class 2 pathogens and Class 1 pathogens) were selected into the GAM model established for the groups aged 0–5 years and over 20 years. Disease risk increased rapidly with the positive rate of Class 2 pathogens in the 20–50% range in the group aged 0–5 years (Fig. 5). For the group aged over 20 years, disease risk went up with the positive rate of Class 1 pathogens until reaching 5% and then became stable afterwards (Fig. 6).

**Figure 7:**
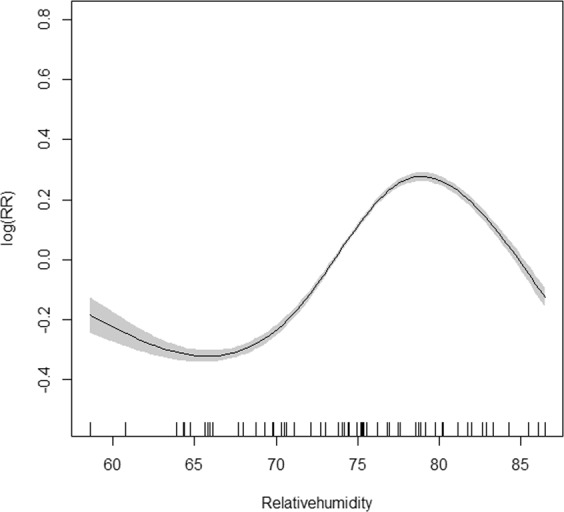
GAM model plot for relatively humidity with infectious diarrhoea in the group aged 0–5 years. (2-month lag).

**Figure 8:**
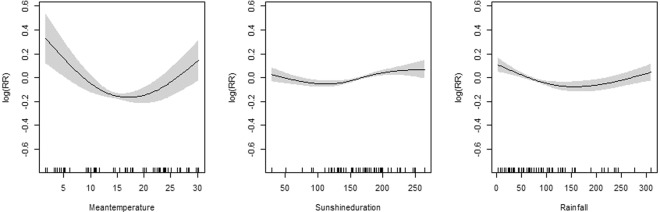
GAM model plot for mean temperature, sunshine duration and rainfall with infectious diarrhoea in the group aged over 20 years.

Relative humidity was introduced into the GAM model for two-month lag morbidity of the group aged 0–5 years. Considering collinearity and goodness of fit, two optimal GAM models were established (one introduced mean temperature and sunshine duration, another introduced rainfall) for synchronous morbidity of the group aged over 20 years (Table 2) (appendix). Disease risk increased with relative humidity between 67–78% and decreased when the relative humidity was too high or too low in the group aged 0–5 years (Fig. 7). The first panel of Fig. 8 depicts the disease risk which decreased to the trough as the mean temperature rose to 15 °C then went up as the mean temperature continued to rise. In the second panel, the disease risk fluctuated around zero when sunshine duration was no more than 150 hours per month and increased afterwards. In the third panel, there was also a U-shape association for rainfall and disease risk, with the threshold of rainfall being 100 mm per month.

## Discussion

This study revealed a long-term increasing trend and distinct seasonality in the morbidity of infectious diarrhoea in Jiangsu Province. Infectious diarrhoea mainly peaked in the winter and became increasingly obvious annually, while the secondary summer peak became weak annually. The trends differ from those observed in other provinces of China^3–5^ and may be due to changes of the pathogen spectrum over time. The seasonality also differed in different populations. Etiological and meteorological factors may influence age-specific incidence of infectious diarrhoea differently. In addition, infectious diarrhoea in Jiangsu Province exhibited an obvious spatial and population distribution. In particular, it was more prevalent in Xuzhou, Yancheng, and the border area between Wuxi and Suzhou. Its prevalence was higher in males and differed largely with age. Infants aged 0–2 years showed the highest morbidity and accounted for nearly 60% of all cases, which is consistent with other reports^3–5,14^. These trends may be explained by the fact that males perform more activities outdoors and have poorer hygiene habits and infants also have weaker immune systems^9,15^, thus warranting strengthened monitoring and interventions (e.g., hygiene education) for these populations, especially in the highly prevalent areas.

Pathogens of infectious diarrhoea were divided into two classes according to their prevalence seasonality (Class 1 and Class 2, were epidemic during June-September and November to next March, respectively). Seasonal variation of morbidity indicated that the epidemic intensity of Class 2 pathogens gradually strengthened, while the epidemic of Class 1 pathogens weakened. Therefore, preventive efforts should be strengthened for Class 2 pathogens in winter. In 2017, the average monthly cases of infectious disease increased and became more pronounced during the winter season. Meanwhile, norovirus had the highest positive rate among all pathogens. This finding may correspond to the numerous norovirus outbreaks in Jiangsu during the same year^16,17^. Attention should be paid to diarrhoea outbreak disposal to prevent the spread of pathogens.

The disease incidence among the group aged 0–5 years rose from November to March showing a similar seasonality with the activity of Class 2 pathogens. Correlation analysis also showed a significant association between the incidence and the prevalence of Class 2 pathogens. Moreover, results of the GAM model suggest that higher positive rate (20–50%) was associated with higher disease risk. This association was not obvious at first but became apparent with an exponential increase in the pattern of human-to-human transmission, which eventually grew steadily because the population gained immunity^18^. In addition to the faecal-oral route, the major pathogens in Class 2 such as rotavirus and norovirus can be transmitted among children through airborne droplets and vomit^19,20^. Since the family planning policy was cancelled in 2013, the number of Chinese children has drastically increased^21^. Among them, those aged 0–5 years spend more time indoors, which increases the possibility of human-to-human transmission. Moreover, the mean temperature, relative humidity, temperature range, and rainfall were all related to morbidity by two-month lag in the group aged 0–5 years. Of these, relative humidity showed the strongest correlation with morbidity. The GAM model showed the risk of disease increased with relative humidity between 67–78%, which is consistent with previous studies^22^. Appropriate relative humidity facilitates pathogen growth and airborne transmission.

The incidence among the group aged over 20 years peaked in July-August. The prevalence of Class 1 pathogens, mean temperature, sunshine, and rainfall were strongly related to morbidity. The risk of infectious diarrhoea rose gradually with the prevalence of Class 1 pathogens at a low level. Bacterial pathogens trigger infectious diarrhoea through ubiquitous faecal-oral transmission. This low level may be associated with immunity developing from frequent exposure to antigens^23–25^. Both heat and cold can increase the risk of infectious diarrhoea, which may be partially explained by the following reasons. First, higher temperature may increase the intake of water and raw food; once contaminated, this intake may promote bacterial or viral transmission. Second, heat and cold may distort the immune and intestinal system. Research shows that adrenocortical activity and serum immunoglobulin levels decrease as ambient temperature increases. In the winter, elevated adrenocortical activity depresses T-cell function^26,27^. Third, high temperature can prolong the survival of bacteria in food, such as *Escherichia coli*^28,29^. Fourth, in a hot air, some pathogenic organisms such as plankton-carrying microbes proliferate faster in warm water. Longer sunshine duration (more than 150 hours per month) also increases the risk of infectious diarrhoea. A study conducted in Lanzhou of China found that the number of infectious diarrhoea cases increased by 6.39% per day, with an inter-quartile range determined by daily sunshine duration^30^. Another study showed that salmonellosis, vibriosis, and *E. coli* O157:H7 infections correlated positively with insolation^31^, which is consistent with our results. Both drought and humidity can increase the risk of diarrhoea. Due to the lack of rainfall, safe drinking water and sanitation would be deteriorated. In a region with rich rainfall, waterborne pathogens can be transmitted easier^32–34^, thereby increasing the risk of infectious diarrhoea. Thus, measures to safeguard water quality are necessary.

In the group aged 6–19 years, infectious diarrhoea occurred in multiple months and presented two seasonal peaks in December and March. However, no significant associations were detected between disease prevalence and etiological and meteorological factors. Two main reasons may account for this. First, the cases in this age group only accounted for 11.71% of all cases, making it hard to detect the association. Second, a variety of pathogens were detected in this age group. It can be concluded that the pathogen spectrum in Jiangsu is so wide that the relationship between a single class pathogen’s prevalence and meteorological factors could not be found.

This study has some limitations. First, not all of the infectious diarrhoea cases were validated in the laboratory, so we do not have complete etiological data. Second, people with mild infectious diarrhoea may not seek medical care and remain unreported, leading to biases in the incidence of the disease.

In summary, we described the epidemiological characteristics of infectious diarrhoea and identified the basic components of the infectious diarrhoea pathogen spectrum in Jiangsu Province. It is beneficial to carry out targeted prevention and control and pathogen monitoring in high-incidence populations and areas. We also comprehensively illustrated the age-specific effects of etiological and meteorological factors on infectious diarrhoea, which are crucial to predict morbidity and develop adaptation strategies.

## Methods

### Study area

Jiangsu Province is located in the eastern part of China (latitude 30°46′–35°08′N and longitude 116°21′–121°54′E), consisting of 13 cities (108 counties) and a population of about 80 million.

### Data source

In China, infectious diarrhoea (excluding cholera, dysentery, typhoid, and paratyphoid) is an intestinal infectious disease with diarrhoea and/or vomiting as the main symptom. Data on monthly infectious diarrhoea morbidity during 2013–2017 in Jiangsu Province were collected from the Nationwide Notifiable Infectious Diseases Reporting Information System (NIDRIS)^35^. The information included gender, age, address, and date of onset.

Through multi-stage sampling, we selected 26 hospitals to examine the prevalence of infectious diarrhoea from Wuxi, Xuzhou, and Nantong (cities of Jiangsu). Stool specimens were collected to conduct bacteria culture and virus nucleic acid detection (Real-time Polymerase Chain Reaction, Real-time PCR). We tested the level of rotavirus, norovirus, sapovirus, adenovirus, astrovirus *Vibrio parahaemolyticus*, *Salmonella*, *Escherichia coli* and *Campylobacter jejuni* in each sample.

Monthly climate data were obtained from the National Meteorological Information Center (http://data.cma.cn/). It included mean temperature, temperature range, relative humidity, sunshine duration, and rainfall.

### Data analysis

Descriptive statistics was employed to illustrate the epidemiological characteristics of infectious diarrhoea, etiological, and meteorological factors. Seasonal decomposition was applied to analyse incidence time series. Ordinary kriging interpolation^36^ was employed to spatially visualize disease incidence. Seasonal index (SI)^37^ was calculated to signify the seasonality of infectious diarrhoea in various populations and pathogens. Based on the SI, agglomerative hierarchical algorithm^38^ was employed to aggregate various population and pathogens and further represented data as thermodynamic diagrams. Formulas are specified as follows:

$${\bar{x}}_{j}=\frac{{\sum }_{i=2013}^{2017}{x}_{ij}}{5}$$

$$\bar{x}=\frac{{\sum }_{i=2013}^{2017}{\sum }_{j=1}^{12}{x}_{ij}}{60}$$

$$S{I}_{j}=\frac{{\bar{x}}_{j}}{\bar{x}}$$

*i*represents year from 2013 to 2017, *j* represents month from January to December, *x*_*ij*_ represents the number of cases (or pathogen positive cases) in month *j* of year *i*, ${\bar{x}}_{j}$ represents the average cases (or pathogen positive cases) of month *j* during 2013–2017, $\bar{x}$ represents the monthly average cases (or pathogen positive cases) during 2013–2017, *SI*_*j*_ represents the *SI* of month *j*.

Cross-correlation analysis was then used to measure the association between the etiological and meteorological factors and zero to two monthly lagged morbidity in each age group. To assess for potential multicollinearity of meteorological factors, Spearman’s correlation coefficients (r_s_) were also calculated between climate factors. If the absolute value of r_s_ (i.e. |r_s_|) between related meteorological factors was >0.7, separate models were considered for subsequent quantitative analysis^39,40^. The optimal model was selected based on Akaike Information Criterion for quantitative analysis of factors.

Finally, the GAM model was employed to quantify the age-specific effect of highly correlated etiological and meteorological factors (|r_s_| > 0.4 and *P* < 0.05) on infectious diarrhoea^8^. Many studies have shown that meteorological factors also play a role in aetiology^11,12^, so different GAM models were established to access the effect of etiological or meteorological factors on infectious diarrhoea in various age groups to avoid potential confounding. According to the Kolmogorov-Smirnov test, age-specific morbidity in this study conformed to the Poisson distribution (*Z*_*0–5*_ = 5.67, *P*_*0–5*_ < 0.01; *Z*_*6–19*_ = 5.67, *P*_*6–19*_ < 0.01; *Z*_20*-*_ = 3.32, *P*_*20-*_ < 0.01) and there were no signs of overdispersion based on the overdispersion test (*D*_*0–5*_ = 1.24, *P*_*0–5*_ = 0.65; *D*_*6–19*_ = 1.66, *P*_*6–19*_ = 0.44; *Z*_*20-*_ = 1.22, *P*_*20*_ = 0.71). Therefore, log was selected for the link function. Penalized smoothing spline was used to adjust for long-term trends, seasonality, and related factors. Degrees of freedom (df) was based on generalized cross-validation. The GAMs are specified as follows:

$$\log [E({Y}_{t})]=\alpha +s(tl,df)+s(ts,df)+s(meteorological\,variables,df)$$

$$\log [E({Y}_{t})]=\alpha +s(tl,df)+s(ts,df)+s(etiological\,variables,df)$$

*Y*_*t*_ represents the monthly number of cases in various group, *E*(*Y*_*t*_) represents the expected value of *Y*_*t*_, *α* represents the model intercept, *s*() represents penalized smoothing spline, *tl* represents time to control long-term trend, *ts* represents month to control seasonality

The methods above were performed in R (version 3.5.1). The “mgcv” package in R was used to fit the GAM model. Statistical significance was defined as *P* < 0.05. ArcGIS (version 10.4.1) was used for the ordinary kriging interpolation.

### Ethics approval and consent to participate

This study was part of diarrhea surveillance in Jiangsu Province. The study protocol has been viewed and approved by the Ethics Committee of Jiangsu Provincial Center for Disease Control and Prevention and all experiments were performed in accordance with relevant guidelines and regulations. Informed consents have been obtained from all participants or their legal guardians if participants were under 18 years old.

## Supplementary information

The basic information on meteorological factors and GAM models

## Data Availability

The datasets generated in this study are available from the corresponding author on reasonable request.
