# Supplementary material for: Epidemiology of infectious diarrhoea and the relationship with etiological and meteorological factors in Jiangsu Province, China
Source: Sci Rep. 2019 Dec 20;9:19571. doi: 10.1038/s41598-019-56207-2 (PMC6925108; doi:10.1038/s41598-019-56207-2)
Supplement: Supplementary file 1 — The basic information on meteorological factors and GAM models [file 41598_2019_56207_MOESM1_ESM.docx]

## Epidemiology of infectious diarrhoea and the relationship with etiological and meteorological factors in Jiangsu Province, China

Xinyu Fang^1,2#^, Jing Ai^2#^, Wendong Liu^2#^, Hong Ji^2^, Xuefeng Zhang^2^, Zhihang Peng^1^, Ying Wu^2^, Yingying Shi^2^, Wenqi Shen^2^, Changjun Bao^1,2,3*^

1. School of Public Health, Nanjing Medical University, Nanjing, 211166, China.

2. Jiangsu Provincial Center for Disease Control and Prevention, Nanjing, 210009, China.

3. NHC Key laboratory of Enteric Pathogenic Microbiology, Nanjing, 210009, China.

# These authors contributed equally to this work.

* Corresponding author: Email: bao2000_cn@163.com, phone number:02583759404, Fax: +862583759409.

**Supplementary Information**

Table 1. The basic information on meteorological factors in Jiangsu Province, 2013-2017

|  | Mean±SD | Percentiles | | | | |
| --- | --- | --- | --- | --- | --- | --- |
|  |  | Min | P25 | P50 | P75 | Max |
| Mean temperature（°C） | 15.84±8.85 | 1.65 | 8.47 | 16.77 | 23.67 | 30.21 |
| Relative humidity（%） | 74.36±60.67 | 58.59 | 69.84 | 75.07 | 78.93 | 86.45 |
| Temperature range（°C） | 8.34±1.33 | 5.15 | 7.36 | 8.15 | 9.36 | 11.64 |
| Sunshine duration（h/month） | 156.26±55.33 | 30.00 | 131.00 | 161.00 | 190.70 |  |
| Rainfall（mm/month） | 93.66±74.00 | 3.70 | 35.16 | 72.96 | 128.71 | 309.08 |

Table 2. Parameters in GAM models for infectious diarrhea in Jiangsu Province, 2013-2017

| Models | AIC | tl | | ts | | Etiological and meteorological variables | | |
| --- | --- | --- | --- | --- | --- | --- | --- | --- |
|  |  | Estimated  degrees of  freedom | *P* | Estimated  degrees of  freedom | *P* | Variable | Estimated  degrees of  freedom | *P* |
| log[E(Y_0-5_)] =α+s(tl)+s(ts)+s(Class2 pathogens) | 6167.05 | 2.96 | <0.01 | 2.98 | <0.01 | Class2 pathogens | 2.98 | <0.01 |
| log[E(Y_20-_)] =α+s(tl)+s(ts)+s(Class1 pathogens) | 1236.80 | 2.85 | <0.01 | 2.98 | <0.01 | Class1 pathogens | 2.93 | <0.01 |
| log[E(Y_0-5_)] =α+s(tl)+s(ts)+s(relative humidity) | 6562.56 | 3.00 | <0.01 | 3.00 | <0.01 | relative humidity | 3.00 | <0.01 |
| log[E(Y_20-_)] =α+s(tl)+s(ts)+s(mean temperature)  +s(sunshine duration) | 896.35 | 2.85 | <0.01 | 3.00 | <0.01 | mean temperature | 2.92 | <0.01 |
|  |  |  |  |  |  | sunshine duration | 2.89 | <0.01 |
| log[E(Y_20-_)] =α+s(tl)+s(ts)+s(rainfall) | 1152.77 | 2.96 | <0.01 | 3.00 | <0.01 | rainfall | 3.00 | <0.01 |
| log[E(Y_20-_)] =α+s(tl)+s(ts)+s(rainfall)  +s(sunshine duration) | 1048.59 | 2.73 | <0.01 | 2.98 | <0.01 | rainfall | 3.00 | <0.01 |
|  |  |  |  |  |  | sunshine duration | 2.68 | <0.01 |

Note: Y_0-5_ and Y_20-_ represent the monthly number of cases in 0-5 years age group and over 20 years age group respectively, E(Y_0-5_) and E(Y_20-_) represent the expected value of Y_0-5_ and Y_20-_, α represents the model intercept, tl presents time to control long-term trend, ts represents month to control seasonality
